# The primary reasons behind data sharing, its wider benefits and how to cope with the realities of commercial data

**DOI:** 10.1186/s12864-015-1789-5

**Published:** 2015-09-07

**Authors:** Ross L. Tellam, Paul Rushton, Peter Schuerman, Irene Pala, Derek Anane

**Affiliations:** CSIRO Agriculture, Queensland Biosciences Precinct, 306 Carmody Rd, St Lucia, QLD, 4067 Australia; Texas A&M AgriLife Research and Extension Center, Dallas, Texas USA; University of California, Merced, CA USA; BioMed Central, London, UK

## Abstract

Data availability expectations have changed over the years in scientific publishing, nowhere more so than in the field of genomics. This field has spearheaded openness and transparency via public and structured deposition of data. *BMC Genomics* strongly encourages deposition and unrestricted availability of all primary data underlying research studies both as a way of ensuring reproducibility and standardisation, but also as part of overall community-driven expectation on data deposition and sharing.

With funders and publishers moving towards more explicit mandates (regarding data availability), we examined the current barriers to unrestricted availability of data and explored different scenarios in which commercial agreements might run contrary to scientific convention and data sharing policies. In this editorial, Ross Tellam (CSIRO, Australia), Paul Rushton (Texas A&M AgriLife Research) and Peter Schuerman (University of California, Merced), give their views on the importance of data sharing and examine the current challenges in research fields like crop and livestock genomics, where often it is necessary to integrate the interests of academic and commercial stakeholders. We discuss the current approaches, highlight the importance of community-driven standards, and propose ways forward.

## Why is providing access to data crucial?

**RT -** Scientific knowledge is cumulative and best used to benefit all. This philosophy is deeply imbedded in our societies as knowledge is the building foundation of each generation and ultimately knowledge growth is the inheritance of future generations. The publication of scientific data is a long established and principal means of dissemination of new knowledge and it also provides a link to underpinning scientific principles that have withstood the test of time. This process allows a wider group of scientists to replicate and then importantly, extend knowledge in unanticipated directions. In the past, data underwent minimal processing before it was published and it could be easily replicated by others based on information contained solely within the body of the publication. This is now changing.

**PR, PS -** When reviewing a new manuscript for possible publication, a reviewer’s job is to assess the presented data and make a recommendation on publication based on the data itself and the author’s interpretation of the meaning of that data. So, it appears self-evident that all data should be presented so that it can be accurately assessed. Has the data been analysed correctly? Are the conclusions drawn by the authors valid? Could the experiments be repeated by an independent research group and the data directly compared? Is the data in such a form that it can later be used as the basis of comparisons with other data sets? Is there any possibility of academic fraud?

However, the reality of data sharing is not so black and white. Authors may see disadvantages in releasing large data sets that have not yet been extensively analysed that could be further used by rival scientists. Commercial sponsors may not wish to release large data sets because they view the research sponsorship as an investment, and giving competitors access diminishes or eliminates their return on investment.

## Why is data sharing such an issue at present?

**RT -** Knowledge is rapidly increasing and evolving. This is being driven by the scientific desire to address the complex questions of nature and is coupled with markedly enhanced technical capability often involving micro-scale analyses performed in massively parallel arrays in multiple experimental dimensions intersecting with increased computational and statistical capacities. Genetics and genomics are at the forefront of these changes [1].

Science is also moving from deterministic to more probabilistic investigations. The former is exemplified in genetics by a mutation of large effect where the presence of the mutation in an individual causes a phenotype such as a specific disease or developmental malformation. The probabilistic approach is typified by population genetics and multigenic traits, such as metabolic and disease resistance traits, where a large number of interactive genotypes each of small effect size contribute to an increased risk of acquiring the trait in the population but not necessarily in an individual. The scale of data generation in this latter instance is large particularly for genomics technology.

## What are the challenges to data deposition?

**RT -** The life sciences now face unique challenges in relation to public access to ‘omics data. First, the sheer quantity and scale of data is a practical issue in terms of data transfer, storage, retrieval and maintenance [2]. However, large public access databases have been established for most ‘omics technologies although these are not universally used by scientists. The databases facilitate the deposition and internet-based redistribution of data. They also provide considerable benefit to data generators who, with time, invariably want to free up computational storage space and guard against accidental data loss. Large datasets are also costly to generate and often have unrealised scientific value. Hence, new collaborations and new research opportunities are facilitated by data deposition as few large data sets are analysed to their full potential. For some databases a depositor can also be provided with a period of exclusive use of the data thereby allowing them to maximally benefit from their efforts.

## What has been the general approach to address problems with data deposition?

**RT-** The Human Genome Project, in its very early stages, recognized the importance to the scientific community of large-scale data deposition in public access facilities while also preserving the rights of the data generators to analysis and publication of their data in a holistic fashion. This model has served us well and indeed it has been the catalyst for enhanced national and international collaborations resulting in massive increases in genomic and genetic knowledge over the last 25 years as well as new commercial enterprises [3–5].

**PR, PS -** Guided by the principle of peer review, and to address the hesitation in releasing large data sets, both federal agencies and journals have established policies on data sharing. While policies differ, in general, there is currently a move towards making data available as a condition of publication. Some publishers are moving towards mandating that all underlying data should be shared. For example, *Science* instructs authors that “All data necessary to understand, assess, and extend the conclusions of the manuscript must be available to any reader of *Science*” and goes on to add “appropriate data sets… must be deposited in an approved database.” However, these instructions are subject to interpretation. Does the word “appropriate” mean that only a very small portion of a large omics data set need be made available? After all, only a small fraction is discussed in any detail. Alternatively, does the expression “all necessary data” require that the large data set be deposited in its entirety?

## Do you think mandating the sharing of data is appropriate?

**PR, PS –** While it’s encouraging to see journals moving in this direction and mandating data deposition [6] there can be difficulties in explaining what is exactly required [7, 8]. Increasingly there will be a trend for journals to make data available as a condition of publication but the devil is in the detail and the rules are often unclear.

## Is there a “halfway” approach to data deposition?

**PR, PS –** A different approach is being taken by *Scientific Data* [9]. Scientific Data aims to “address the increasing need to make research data more available, citable, discoverable, interpretable, reusable and reproducible.” Rather than mandating data sharing, this approach seeks to reward scientists for releasing their data, and ensures data quality adheres to community standards. The incentives for scientists are that they produce a publication in a peer reviewed journal and that they don’t have to present any findings from the data, just the data itself. The review process ensures that the data is of high quality and in alignment with community standards, so other users of the data also benefit.

*GigaScience and F1000 Research have spearheaded this trend since 2012, as a response to increasingly data-driven research. In GigaScience, standard scientific publishing is linked directly to a database that harbours all the relevant supporting data in a citable format.*

## What about sharing proprietary data, under license agreements that limit its use?

**PR, PS -** Another layer of complexity is created when researchers collaborate with companies. Data generated in collaborative or sponsored research may have strategic benefits for the commercial partner, and releasing a data set into the public domain may not be in their best interests.

No uniform approach or policy for data sharing is appropriate to such collaborations. The solution lies in first considering the needs of both parties regarding the disposition of the data – for example, the university’s need to publish and the company’s need to get a return on their investment. Next, creativity is required in balancing those needs. While this is simple to say, in practice this can be a challenge, unfortunately most universities have historically repurposed their federal contracting infrastructure to work with industry, which treats deal-making like forms-processing and eliminates the opportunities for creativity.

For example, at the outset of a collaboration discussion, a company that is sponsoring research may want to own all of the data that results from the research. The investigator, however, may have different goals such as publishing in a particular journal or set of journals. The compromise may be to define certain categories of data as having different sensitivities, or to de-identify certain types of data, or to coordinate on patent filings before releasing data – with the result being that both publication and proprietary benefit are achieved. Unless we make room in our discussions for this type of creative problem-solving and compromise, we run the risk of failing to nurture relationships between academia and industry.

**RT-** Many genomics technologies are now being applied to commercially important agricultural populations, in which elite germplasm is the primary commodity of value [10]. Consequently there is strong reluctance of data generators to place genotypes in the public domain as these directly relate to their commercial interests. It is interesting to note that these commercial activities have frequently grown out of genome sequencing projects, indeed these commercial initiatives are one of the great successes of these largely publicly-funded enabling projects [11]. The argument now emerging in some quarters, that these commercial interests outweigh the need for primary data deposition associated with scientific publication, is not logical or strong. There is no benefit to science if publications do not reveal primary underpinning data as the study cannot be verified by replication and cannot be used to direct science in new directions. The proposed scientific publication should be subject to the normal standards associated with data deposition and public access. If there is commercial value in ‘omics data, especially genotype data, then it can be held in private by the commercial interests, although this precludes public benefit. One compromise is for databases to guarantee a limited period of exclusivity to the data depositor. This model recognises the important contributions of investigators and ensures that commercial data do eventually become publicly available for wider analysis. The relevant parties at the commencement of the research should also discuss the interplay between commercial reality and the freedom to publish and then identify a pathway for mutual benefit. This is especially important for the careers of young scientists on short term contracts funded by industry.

## Is data privacy a major issue for human data?

**RT -** Genotype data not only defines the individual it also can define relatives and therefore agreements with one party for public deposition of genotype information can impact on other parties who have not agreed to public release [12]. Hence, human data should be deposited in databases where there is controlled access and controlled conditions of use. Importantly, these databases often have tiered levels of access that provide different levels of confidentiality and control which can be nominated by the data depositor and therefore are well-suited to the deposition of human data underpinning publications. It should be noted that the all pervasive use of DNA sequencing technology for many experimental purposes allows genotype to be deduced from indirect sources e.g. via RNA-seq, Me-seq and ChIP-seq. These possibilities reinforce the view that all human sequence information in the public domain should not be linked to individual identifiers.

## How can funding agencies, publishers, and data repositories help address these issues?

**PR, PS -** Universities are being asked to become more engaged with companies and to translate more of their research results into economic growth and job creation. However we are also seeing new policies on data sharing which may place limits on the creativity that can be deployed in creating win-win arrangements. As we work to balance our interests of promoting science and economic development, broad-brush data sharing rules and policies have the potential to hinder both. Federal agencies and journals have a responsibility to consider their rules and policies in this broader context.

*Open data projects, such as the Human Genome Project have had a substantial impact on the creation of jobs and a massive influence on the economic returns to private industry* [13]*. On a broader context, non-commercial initiatives such as the generation of GPS data provided clear benefits to companies and spurred economic growth that would not have been possible under restricted-use data deposition.*

**RT -** The international scientific community should initially reach agreement on the minimum annotation and quality standards for data deposition and then support long term funding for public access databases. Ideally, the databases should not only store and distribute data but also provide flexible analytical tools to promote reanalysis. By structuring access, various levels of confidentiality and time limited exclusive use can be made available thereby removing barriers preventing data from emerging into the public space. There are also ethical responsibilities of users of the deposited data that should be clearly articulated. Many databases are already well established in this regard and indeed are leading policy development in these areas.

Scientific publication is an efficient means of knowledge dissemination but there should be encouragement of authors to place large scale datasets into the public domain. The scientific community, public and data contributor will all benefit. Of course, the real scientific achievement is the conversion of these massive data quantities into new knowledge. This is not an easy task as there are few all encompassing conceptual frameworks for data analysis in biology. The more people who investigate these large datasets, the more likely these new concepts will emerge – biological research needs them.

*Some community-driven initiatives are already in place to tackle quality standards in data deposition and curation and this trend is being supported and encouraged by funders, with increasingly stronger mandates for open data* [14]*. The Genomic Standards Consortium (**http://gensc.org/**) has been formed to directly address the issues of transparency and to develop a standardized approach to data capture and exchange* [15]*. The ENCODE project has also released minimum standards for many genomics technologies* [16]*.*

At *BMC Genomics* we believe that our data availability policy should be led by researchers working within a particular speciality. As such we recognise the importance for researchers across genomics to have access to data underlying studies published in our journal. We therefore continue to strongly encourage all authors who submit to our journal to deposit data from their studies in publicly accessible repositories and will continue to reform our policies as the field inevitably edges towards universal accessibility. As always, we very much welcome your views and invite discussion.
